# Multimeric forms of CD40 ligand (CD40L), 4-1BB ligand (4-1BBL), OX40 ligand (OX40L), CD27L/CD70, and other TNFSFs as tools for adoptive cell therapy

**DOI:** 10.1186/2051-1426-1-S1-P20

**Published:** 2013-11-07

**Authors:** Richard S Kornbluth

**Affiliations:** 1 Multimeric Biotherapeutics, Inc., La Jolla, CA, USA

## 

The TNF SuperFamily (TNFSF) of ligands include 19 molecules some of which play key roles in the immune system. All of these ligands are produced as trimeric Type II membrane molecules that can be released from the cell surface as single trimers. However, more than a decade of research has shown that the receptors for TNFSFs on responding cells require clustering in order to signal. Thus, single trimers are poor stimuli because they cannot produce the necessary clustering. Likewise, studies of agonist anti-TNFSF receptor antibodies (anti-CD40, anti-4-1BB, anti-DR5, etc.) have shown that these antibodies must be mounted via their Fc portion to FcRs on an adjacent cell in order to cluster and signal these receptors. This means that agonistic antibodies to TNFSF receptors only function in microenvironments where there is a directly adjacent FcR-bearing cell. However, we have solved the receptor clustering problem by creating fusion proteins that contain many TNFSF trimers. As scaffolds for these molecules, we used surfactant protein D (SP-D) to make molecules with 4 trimeric arms (“UltraLigands™”) and ACRP30 (A Complement-Related Protein 30 kD or adiponectin) to make molecules with 2 trimeric arms (“MegaLigands™”). Numerous papers from independent labs have reported the ability of UltraCD40L™ and MegaCD40L™ to activate human and mouse cells. UltraOX40L™and MegaOX40L™ have also been studied and most recently Ultra4-1BBL™ has been produced. We are applying these unique materials in the following ways: (1) Using MegaCD40L™ or UltraCD40L™ plus IL-4, human B cells have been grown from small amounts of blood to serve as highly efficient APCs for expanding antigen-specific CD8+ T cells in vitro; (2) Mega4-1BBL™ or Ultra4-1BBL™ are being used to activate TCR-stimulated CD8+ cytotoxic T cells in vitro; and (3) MegaOX40L™ or UltraOX40L™ are being used to stimulate CD4+ T cells in vitro. These cell-free proteins can be used as practical reagents to simplify and improve the generation of immune cells for adoptive immunotherapy.

